# Effects of Administration and Intensity of Statins on Mortality in Patients Undergoing Hemodialysis

**DOI:** 10.3390/ph17040498

**Published:** 2024-04-13

**Authors:** Yunmee Lho, Gui Ok Kim, Bo Yeon Kim, Eun Jung Son, Seok Hui Kang

**Affiliations:** 1 Senotherapy-Based Metabolic Disease Control Research Center, Yeungnam University, Daegu 42415, Republic of Korea; ckdwjdgus@naver.com; 2 Quality Assessment Department, Health Insurance Review and Assessment Service, Wonju 26465, Republic of Korea; 3 Healthcare Review and Assessment Committee, Health Insurance Review and Assessment Service, Wonju 26465, Republic of Korea; 4 Division of Nephrology, Department of Internal Medicine, College of Medicine, Yeungnam University, Daegu 42415, Republic of Korea

**Keywords:** hemodialysis, intensity, mortality, statins, survival

## Abstract

(1) Background: Few studies have investigated the association between the intensity of statins and patient survival rates in patients undergoing hemodialysis (HD) as primary outcomes. This study aimed to evaluate patient survival rates according to the intensity of statins using a large sample of patients undergoing maintenance HD. (2) Methods: Data from a national HD quality assessment program were used in this study (n = 53,345). We divided the patients into four groups based on the administration and intensity of statins: Group 1, patients without a prescription of statins (n = 37,944); Group 2, patients with a prescription of a low intensity of statins (n = 700); Group 3, patients with a prescription of a moderate intensity of statins (n = 14,160); Group 4, patients with a prescription of a high intensity of statins (n = 541). (3) Results: Significant differences in baseline characteristics were observed among the four groups. Group 1 had the best patient survival among the four groups in the univariate Cox regression analyses. However, multivariable Cox regression analyses showed that the patient survival rate was higher for Group 3 than for Group 1. Cox regression analyses using data of a balanced cohort showed that, on univariate analyses, the HRs were 0.93 (95% CI, 0.91–0.95, *p* < 0.001) in Group 2 and 0.95 (95% CI, 0.93–0.96, *p* < 0.001) in Group 3 compared to that in Group 1. Group 4 had a higher mortality rate than Groups 2 or 3. The results from the cohort after balancing showed a similar trend to those from the multivariable Cox regression analyses. Young age and less comorbidities in Group 1 were mainly associated with favorable survival in Group 1 in the univariate analysis using cohort before balancing. Among the subgroup analyses based on sex, age, presence of diabetes mellitus, and heart disease, most multivariable analyses showed significantly higher patient survival rates in Group 3 than for Group 1. (4) Conclusions: Our study exhibited significant differences in baseline characteristics between the groups, leading to limitations in establishing a robust association between statin intensity and clinical outcomes. However, we conducted various statistical analyses to mitigate these differences. Some results, including multivariable analyses controlling for baseline characteristics and analyses of a balanced cohort using propensity score weighting, indicated improved patient survival in the moderate-intensity statin group compared to non-users. These findings suggest that moderate statin use may be associated with favorable patient survival.

## 1. Introduction

Hemodialysis (HD) is the most common renal replacement therapy used to treat patients with end-stage renal disease [1,2]. Patients undergoing HD have a lower chance of survival than those without dialysis [3]. Cardiovascular disease is the leading cause of death in patients undergoing HD. The management of traditional risk factors, such as hypertension, hyperglycemia, and dyslipidemia, is considerably associated with the prevention of and reduction in cardiovascular disease and/or death [4]. These traditional risk factors are also important in patients undergoing HD; however, data on their effects on patient survival are scarce compared to those available for the general population. Small sample sizes have made conducting comprehensive and high-quality research challenging. In addition, complex comorbidities in patients undergoing HD cause confounding effects that hinder or overestimate the association between the interventions and their effects. Therefore, some traditional risk factor suggestions are based on data from the general population or insufficient studies.

Inconsistent results exist regarding the administration of statins for treating dyslipidemia in patients undergoing HD, which remains an unresolved issue. Previous observational studies have reported improved outcomes in statin-treated patients undergoing HD [5,6]. However, three randomized trials did not show an association between statin administration and survival rates in patients undergoing HD [7,8,9]. These inconsistent results may be due to the characteristics or intensity of the statins. Some researchers have focused on the hazardous effects of lipophilic statins, which may reduce the benefits of statins [10,11,12,13]. The intensity of statins may also play an important role in the different results regarding the association between statin administration and survival rates in patients undergoing HD. However, few studies have investigated the association between the intensity of statin and patient survival rates as primary outcomes. This study aimed to evaluate patient survival rates according to the intensity of statins using a large sample of patients undergoing maintenance HD.

## 2. Results

### 2.1. Clinical Characteristics

**Figure 1:**
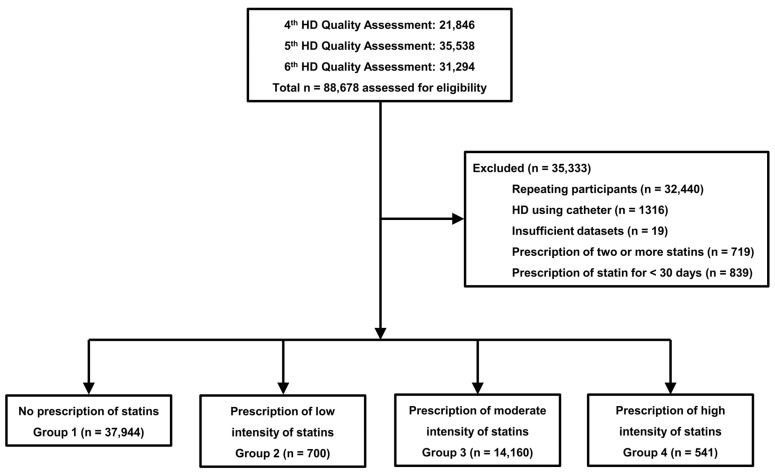
Study flowchart.

Figure 1 shows the flowchart of the participants.

Initially, 88,678 patients were included in this study. Among them, 32,459 patients were excluded because of repeated participation or insufficient data. Additionally, 1316 patients were excluded because they had undergone HD with a non-tunneled or tunneled catheter. Finally, we excluded 1558 patients who were prescribed two or more statins or statins for <30 days during the six months of each assessment. In total, 53,345 patients were included in this study. The numbers of patients in Groups 1, 2, 3, and 4 were 37,944, 700, 14,160, and 541, respectively (Table 1).

Group 1 had a higher proportion of male patients and a lower proportion of diabetes mellitus (DM), myocardial infarction (MI), or congestive heart failure (CHF), and administered renin–angiotensin system blockers, aspirin, or clopidogrel than those in the other groups. Patients in Group 1 had lower Charlson comorbidity index (CCI) scores and were younger than those in the other groups. In addition, the patients in Group 1 had greater HD vintages, follow-up duration, pre-dialysis diastolic blood pressure (DBP), and phosphorus and creatinine serum levels than those in the other groups. Patients in Group 4 had a lower proportion of arteriovenous fistula than the other groups.

### 2.2. Survival Analyses

The number of patients in the survivor, death, peritoneal dialysis, or kidney transplantation subgroups at the end-point of follow-up were 19,995 (52.7%), 14,726 (38.8%), 143 (0.4%), and 3080 (8.1%) in Group 1; 346 (49.4%), 292 (41.7%), 3 (0.4%), and 59 (8.4%) in Group 2; 7758 (54.8%), 5481 (38.7%), 37 (0.3%), and 884 (6.2%) in Group 3; and 283 (52.3%), 228 (42.1%), 3 (0.6%), and 27 (5.0%) in Group 4, respectively (*p* < 0.001).

**Figure 2:**
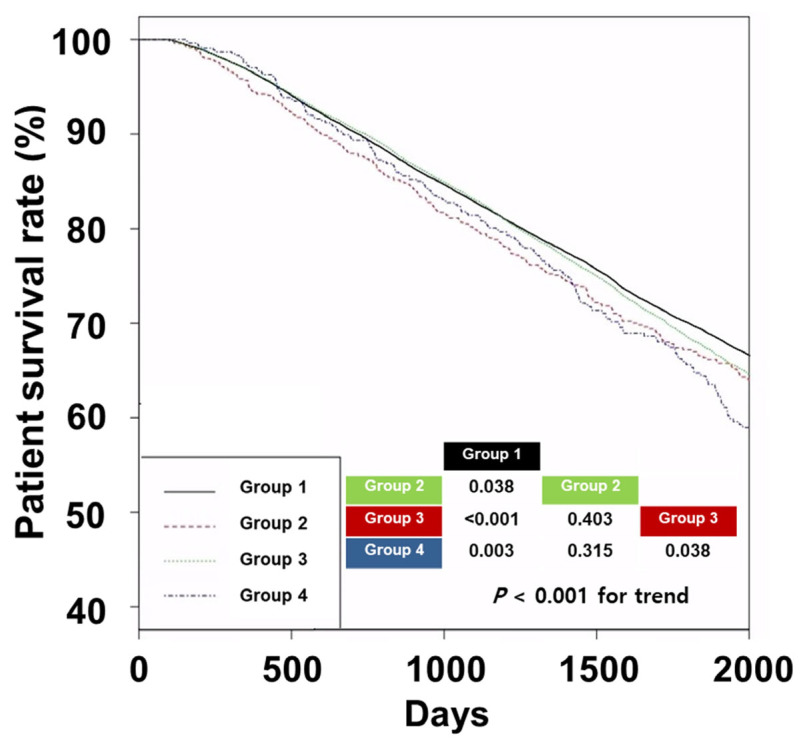
Kaplan–Meier curves of patient survival according to groups. The *p*-values for pairwise comparison or trend with log-rank tests were added to the lower right corner of the graph. Abbreviations: Group 1, patients without prescription for statins; Group 2, patients with prescription for low-intensity statins; Group 3, patients with prescription for moderate-intensity statins; Group 4, patients with prescription for high-intensity statins.

The 5-year survival rates in Groups 1, 2, 3, and 4 were 69.6%, 67.0%, 68.0%, and 64.7%, respectively (shown in Figure 2); *p* < 0.001 indicates a significant trend. 

Group 1 had a higher patient survival rate than that of the other groups, and Group 3 had a higher patient survival rate than that of Group 4. Univariate Cox regression analyses showed that the hazard ratios were 1.14 (95% confidence interval [CI], 1.02–1.28) in Group 2, 1.08 (95% CI, 1.05–1.12) in Group 3, and 1.25 (95% CI, 1.10–1.43) in Group 4 compared to that of Group 1 (Table 2). 

However, multivariable Cox regression analyses did not show statistical significance among the four groups, except when comparing Groups 1 and 3. In multivariable analyses, the patient survival rate was higher for Group 3 than for Group 1. We evaluated the effect of body mass index on mortality in our cohort. Body mass index was a metabolic parameter that is inversely associated with mortality.

Based on baseline characteristics and results of the univariate analysis, we selected variables among various factors where, in Group 1, the values were higher than those in the other groups, with a decrease in the hazard ratio upon an increase in the variables (underlying disease, serum albumin, creatinine, phosphorus, calcium, and diastolic blood pressure), or where the values were lower in Group 1 than in the other groups, with an increase in the hazard ratio when the variables were increased (age, vascular access type, CCI score, use of renin–angiotensin system blocker, clopidogrel, or aspirin, and presence of MI or CHF). The results of the multivariable Cox regression analysis, including the group based on intensity of statins and each variable, are summarized in Table S1. The hazard ratio of Group 1 was reversed by the addition of age or CCI score as confounding factors.

Furthermore, we evaluated the effect of serum ferritin levels on mortality in our cohort. The serum ferritin levels were available for 41,084 of 53,345 patients. The mean serum ferritin levels in Groups 1, 2, 3, and 4 were 268 ± 249, 275 ± 243, 263 ± 274, and 285 ± 255 ng/mL, respectively (*p* = 0.102). Hazard ratios of an increase in 1 ng/mL were 1.00 (95% confidence interval: 1.00–1.01, *p* < 0.001) in the univariate analysis and 1.03 (95% confidence interval: 1.02–1.03, *p* < 0.001) in the multivariable analysis, respectively.

**Figure 3:**
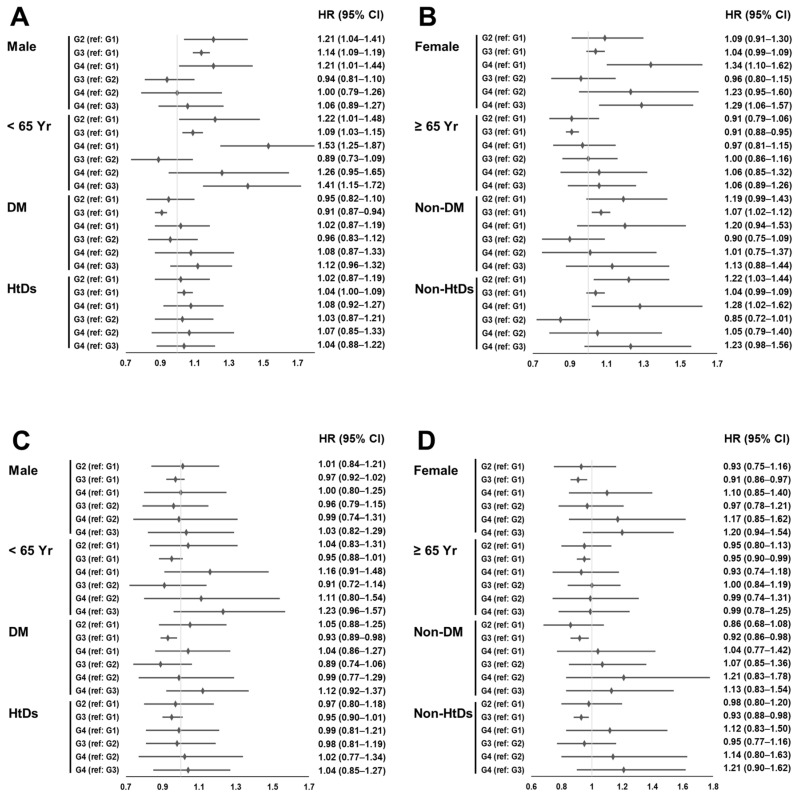
Forest plots of the hazard ratio and 95% confidence interval according to subgroups. (**A**,**B**) Univariate Cox regression analysis; (**C**,**D**) multivariable Cox regression analyses. Adjustment according to age; sex; body mass index; underlying cause of end-stage renal disease; Charlson comorbidity index score; type of vascular access; hemodialysis vintage; ultrafiltration volume; Kt/V_urea_; hemoglobin, albumin, creatinine, phosphorus, and calcium serum levels; systolic and diastolic blood pressure; the use of renin–angiotensin system blockers, clopidogrel, and aspirin; and myocardial infarction or congestive heart failure. Abbreviations: CI, confidence interval; DM, diabetes mellitus; G1, patients without prescription for statins; G2, patients with prescription for low-intensity statins; G3, patients with prescription for moderate-intensity statins; G4, patients with prescription for high-intensity statins; HR, hazard ratio; HtDs, patients with myocardial infarction or congestive heart failure; Non-HtDs, patients without myocardial infarction and congestive heart failure; Yr, years.

We performed subgroup analyses based on sex, age, presence of DM, and heart disease (MI or CHF). Among the subgroup analyses, the multivariable analyses showed significantly higher patient survival rates in Group 3 than in Group 1, except for a statistically non-significant association in male sex, young age, and patients with MI or CHF (shown in Figure 3). However, subgroups with non-significant associations showed trends similar to those with statistically significant associations.

We performed additional analyses on patients with MI or cerebrovascular accidents (CVAs). We defined MI and CVAs based on the CCI criteria. The number of patients with MI in Groups 1, 2, 3, and 4 was 2433 (6.4%), 64 (9.1%), 1649 (11.6%), and 114 (21.1%), respectively. That with CVAs in Groups 1, 2, 3, and 4 was 10,436 (27.5%), 275 (39.3%), 5095 (36.0%), and 244 (45.1%), respectively. The results of the Cox regression analyses are shown in Table 3.

### 2.3. Analyses Using the Balanced Cohort

We compared baseline characteristics and evaluated Kaplan–Meier survival rates using appropriate sampling weights. Balance among the four groups was assessed by calculating the maximum pairwise absolute standardized mean differences (ASMDs) of the covariates before and after balancing (shown in Figure S1). After applying weights, the maximum ASMDs and differences in baseline characteristics decreased for most covariates (Table S2). Kaplan–Meier curves, generated using data of weights, demonstrated that the 5-year survival rates in Groups 1, 2, 3, and 4 were 68.5%, 72.1%, 70.5%, and 69.9%, respectively (shown in Figure S2). Group 3 exhibited higher patient survival rates than did Group 1 (*p* = 0.032).

Cox regression analyses using data of the balanced cohort showed that, on univariate analyses, the HRs were 0.95 (95% CI, 0.93–0.97, *p* < 0.001) in Group 2 and 0.96 (95% CI, 0.94–0.98, *p* < 0.001) in Group 3 compared to that in Group 1 (Table S3). Group 4 had a higher mortality rate than Groups 2 or 3. Multivariable Cox regression analyses demonstrated trends consistent with those of the univariate analyses.

## 3. Discussion

We analyzed 53,345 patients who underwent an HD quality assessment program in the Republic of Korea. Our results revealed that the administration of statins, especially those of moderate intensity, may be associated with improved patient survival. However, the administration of a high intensity of statins diminished the survival benefits of statins. These trends were similar in the subgroup analyses and the analyses using propensity score weighting.

We analyzed the data of the two cohorts, including the total and balanced cohorts. First, we performed analyses using the total cohort. In the univariate analyses, Group 1 had a higher patient survival rate than the other groups, and Group 3 had a higher patient survival rate than Group 4. However, multivariable analyses showed higher survival rates in Group 3 than Group 1. Contradictory results between univariate and multivariable analyses of the association between Groups 1 and 3 were correlated with lower comorbidities in Group 1 than Group 3. These trends were also shown in the results obtained using the subgroups. Next, we analyzed the data using the balanced cohort to reduce confounding factors. These data showed that Groups 2 and 3 had higher patient survival rates than Groups 1 and 4. Patients who were administered a low or moderate intensity of statins, especially moderate intensity, had a higher survival rate than those were not. However, administering a high intensity of statins reduced the benefits observed for the other scenarios.

Statins are administered to treat dyslipidemia in the general population. The administration of statins is also efficacious for the primary or secondary prevention of cardiovascular diseases. Statins are widely administered to treat various conditions associated with cardiovascular diseases. However, inconsistent results have been reported on the association between statin administration and survival in patients undergoing HD. Previous observational studies have reported improved outcomes in statin-treated patients undergoing HD. Mason et al. analyzed data from the Dialysis Outcomes and Practice Patterns Study and evaluated 7365 patients [5]. They have shown that the administration of statins was associated with 23% lower cardiac mortality and 44% lower non-cardiac mortality. Seliger et al. analyzed data from the United States Renal Data System Dialysis Morbidity and Mortality Wave-2 study and evaluated 3716 patients [6]. They also have reported lower all-cause and cardiovascular-specific mortality in patients receiving statins. In contrast to observational studies, randomized trials have shown different results. A 4D study enrolled patients with DM undergoing HD and evaluated the efficacy of atorvastatin [7]. The AURORA study enrolled patients undergoing maintenance HD and assessed the association between rosuvastatin administration and patient outcomes [8]. The SHARP study enrolled non-dialysis and dialysis patients with chronic kidney disease (CKD) and evaluated the efficacy of simvastatin with ezetimibe [9]. Unfortunately, these three studies did not show improved patient survival rates in patients treated with statins. A meta-analysis that included 21 randomized trials showed that the administration of statins improved laboratory findings; however, benefits in cardiovascular events or death were not reported [14].

The results from the univariate analysis using the cohort before balancing showed favorable survival in Group 1 compared to Groups 2 or 3, although these trends were reversed in the multivariable analysis or using the cohort after balancing. Therefore, we performed additional Cox regression analyses with the groups based on intensity of statins and one confounding factor to evaluate the main factors associated with the reversed results. Our results revealed that age and CCI score were mainly associated with favorable survival in Group 1 in the univariate analysis using the cohort before balancing. To avoid the effect of this confounding factor, we performed multivariable, subgroup, and cohort analyses after balancing. These results showed consistent trends. In addition, our results revealed that in the subgroups of MI or CVA, Group 3 had lower mortality than Group 1, although this difference was not significant in the MI subgroup. This may be associated with the limited sample size or the definition of MI based on the ICD-10 code. In addition, serum ferritin, as an inflammatory indicator or iron status, showed a positive association with mortality, despite the large exclusion of samples. This association demonstrates a trend similar to that of previous research findings.

Several issues have been cited regarding the lack of benefits of statins in patients undergoing HD. First, patients undergoing HD were prone to more complex lipid abnormalities and statin resistance than the general population [15,16]. Inflammation or uremic conditions in patients undergoing HD lead to increased levels of atherogenic lipoproteins through oxidation or carbamoylation [15]. In addition, these pathologies lead to the activation of intracellular cholesterol synthesis, which is not fully inhibited by statins despite a decrease in plasma cholesterol [16]. Second, previous studies have suggested that satins affect vascular calcification. Chen et al. analyzed 240 patients who underwent dialysis or kidney transplantation [10]. They found a positive association between coronary artery calcification and statin administration, which could have been caused by the inhibition of vitamin K synthesis. In addition, an in vitro study showed that statins can induce smooth muscle cell death and the subsequent accumulation of calcium [11]. Third, the limitations of the design of previous studies and the class effect of statins may be associated with inconsistent results. Three randomized studies with negative results were followed up for a median of 3.8–4.9 years and were identified as a limitation [7,8,9]. A longer follow-up period may be needed to obtain statistically significant results for hard outcomes such as mortality. In addition, the beneficial effects can differ according to statin class. Statins can be divided into two classes, lipophilic and hydrophilic. However, the clinical and/or experimental results for lipophilic statins have been contradictory. The high penetration of lipophilic statins into the cell membrane can be associated with high cardiac toxicity via interference with the metabolism of cardiac myocytes. However, it is also associated with beneficial effects via easy proximity to the phospholipid head groups [12,13]. The association between statin solubility and survival in patients undergoing HD was beyond the scope of this study. Further studies are needed to determine differences in the solubility of statins in patients undergoing HD. Our study showed improved survival rates in patients receiving a low or moderate intensity of statins than in those not receiving statins or with a high intensity of statins. These results could be associated with a combination of two contradictory effects: the benefits from the administration of statins and reduction in survival benefits due to an increased toxicity as the intensity of the statin increases.

Unlike in general patients, studies on the impact of antiplatelet agents on cardiovascular disease or mortality in patients with CKD are complex and inconsistent. In a study examining the effects of aspirin use on primary prevention in non-dialysis patients with CKD, aspirin use did not reduce cardiovascular disease or mortality [17]. A Taiwanese study using nationwide data showed that the use of antiplatelet agents with aspirin, clopidogrel, or ticlopidine in patients on dialysis (>80% of patients on HD) did not reduce cardiovascular events or mortality [18]. An analysis of the multinational DOPPS cohort showed that the use of aspirin in HD patients did not affect cardiovascular or all-cause mortality, whereas other antiplatelet agents, such as clopidogrel, ticlopidine, dipyridamole, and pentoxifylline, increased cardiovascular and all-cause mortality [19]. In a meta-analysis, benefits were shown in non-fatal/fatal MI for patients with CKD and not on dialysis, and neutral effects were observed in patients on HD. Meanwhile, mortality effects showed neutral effects in patients with CKD who are not on dialysis and those who are on HD [20].

In our study, the results showed a hazard effect of clopidogrel on mortality in the both univariate and multivariable analyses, although the hazard effect of aspirin was observed only in univariate analysis. In addition, significant differences in the baseline characteristics and data on the purpose of antiplatelet agent usage were lacking. Moreover, we did not analyze the cause of the high mortality rate associated with clopidogrel use, and our study had a retrospective observational design. These factors would lead to challenges in drawing clear conclusions regarding the survival effects of antiplatelet agents. Therefore, additional analyses are needed in the future, considering factors such as the timing of intake, underlying conditions, purpose of usage, and additional outcome events such as MI, stroke, and laboratory findings. Additionally, our study showed an inverse association between increases in Kt/V_urea_, hemoglobin, serum albumin, body mass index, blood pressure, and mortality, as well as high mortality rates in arteriovenous grafts as vascular access, which aligns with previous research findings [21]. The contradictory results of an inverse association between phosphorus levels and mortality in the univariate analysis and a positive association in the multivariable analysis may be attributed to changes in the adjustment for nutritional indicators.

In previous randomized controlled trials, moderate-intensity statins effectively decreased low-density lipoprotein (LDL) cholesterol or C-reactive protein levels; however, the use of moderate-intensity statins in patients on HD has demonstrated overall non-significant results on clinical outcomes [22]. However, they may be effective in patients with a high CV risk, such as those with diabetes or high LDL levels [7,8,22,23,24]. In the AURORA study, which focused on patients on HD, rosuvastatin significantly lowered LDL levels, although this did not translate into improved outcomes [8]. However, a post hoc analysis involving patients with DM showed reduced fatal and non-fatal cardiac events [23]. Similarly, a 4D study targeting patients with diabetes on HD failed to impact outcomes initially, although subsequent analysis revealed reduced fatal and non-fatal cardiac events and overall mortality in those with LDL levels of >145 mg/dL, indicating a high risk [7,24]. Our cohort had a longer HD vintage than that in these studies (especially compared to the 4D study) and a higher proportion of patients with diabetes compared to AURORA, which could partly explain why patients with moderate-intensity statins showed better outcomes than non-statin users in our study. Additionally, although LDL cholesterol levels were not available in our study, relatively unfavorable outcomes in non-statin users may be associated with high LDL levels in these patients.

Our study did not include the timing of the statin administration. However, the timing of statin administration may not significantly affect statin-related outcomes. Although evidence regarding the dialyzability of statins is insufficient, most statins are eliminated through fecal excretion [25]. In addition, >80–90% of most statins, except pravastatin, exist in protein-bound form. These results suggest that statin administration was not significantly affected by HD in terms of concentration. A previous study on atorvastatin demonstrated that its concentration and effects were not influenced by HD, leading most clinicians to prescribe statins according to the recommended timing for general intake, regardless of the HD session [26]. However, the potential effects of drug–drug interactions should be considered.

Previous guidelines have recommended that in non-dialysis patients, the LDL cholesterol target should be maintained at <55 mg/dL, with a reduction in LDL cholesterol by ≥50% from baseline for both primary and secondary prevention in very high-risk patients [27]. However, the direct application of these numerical targets or statin use to patients on HD is challenging. The evidence supporting the effectiveness of statins in reducing LDL cholesterol levels in HD patients compared to the general population is limited, and the impact of maintaining LDL cholesterol target levels on cardiovascular disease or mortality in HD patients is unclear [7,8,28,29]. This is attributed to several factors, including the influence of non-traditional risk factors in HD patients on cardiovascular disease incidence, the lower efficacy of statins due to increased levels of high atherogenic lipoproteins such as oxidized LDL or proatherogenic high-density lipoprotein cholesterol in patients on HD, and the difference in the nature of dyslipidemia due to inflammation or malnutrition, making the measured LDL cholesterol levels less reflective of cardiovascular disease risk than in the general population [30]. Nevertheless, previous randomized trials have demonstrated that baseline LDL cholesterol levels range from 100 to 127 mg/dL on average, and moderate-intensity statin use results in a reduction of 32.5% to 42% on average, suggesting a similar trend in patients using moderate-intensity statins in our cohort [7,8,27]. Additionally, because our study lacked cholesterol level data, analyzing the attainment of the target levels was not feasible. However, previous randomized trials did not show evidence of target cholesterol levels, and analyses of target attainment were often omitted owing to insufficient evidence, with many studies focusing solely on usage analysis. Overall, evidence regarding the reduction in cholesterol levels, attainment of target levels, and the subsequent clinical effects of drug use beyond simple statin use in patients on HD is generally lacking, highlighting the need for additional research to establish evidence in this area.

Our study had some limitations. First, our study was retrospective, and the sample size and baseline characteristics of the four groups differed significantly. Second, comorbidities, including heart disease and statin administration, were evaluated using claims data. There may be a discrepancy between actual administration and physician prescriptions. In addition, our study lacked laboratory data on lipid status and etiology of statin administration. Statin effects may differ based on the underlying medical condition they are used for (e.g., MI/CHF vs. dyslipidemia). Third, our data lacked information on the cause-specific mortality, as well as crucial details about heart function, such as heart rate and left ventricular wall thickness (hypertrophy); therefore, knowing more about cardiovascular deaths and these specific heart function measures would have allowed us to go beyond simply analyzing overall mortality and potentially identifying how statin intensity influences patient outcomes differently based on their underlying condition. We utilized real-world data and employed a relatively large sample size, providing our study with the advantage of analyzing the prognosis associated with statin use in patients on HD. However, considering the numerous limitations we encountered, our study is perceived more as a preliminary investigation that can help anticipate considerations before conducting confirmatory studies such as randomized trials. Additionally, the results of this retrospective study highlight the necessity of addressing unresolved issues, thus aiding in the progression toward conducting research that yields clear conclusions.

## 4. Materials and Methods

### 4.1. Data Source and Study Population

Laboratory and clinical data from a national HD quality assessment program and claims data from the Health Insurance Review and Assessment (HIRA) of South Korea were used in this retrospective study [31,32]. The fourth, fifth, and sixth HD quality assessment programs were conducted over a period of 5 years. The fourth program was conducted between July 2013 and December 2013, the fifth program was conducted between July 2015 and December 2015, and the sixth program was conducted between March 2018 and August 2018. The programs included patients who had been undergoing maintenance HD for at least 3 months, who were receiving HD at least twice a week, and who were at least 18 years old. We analyzed the HD quality assessment and claims data of all patients who had undergone HD quality assessment. 

Among them, we excluded patients who were repeat participants, had insufficient data, or underwent HD using a non-tunneled or tunneled catheter. Finally, we excluded patients who were prescribed two or more statins or those who were prescribed statins for <30 days during the six months of each assessment. The Institutional Review Board of Yeungnam University Medical Center approved this study (approval no. YUMC 2022-01-010). Patients did not provide informed consent because their records and information were anonymized and de-identified before the analysis. All methods were performed in accordance with the Declaration of Helsinki and relevant guidelines/regulations.

### 4.2. Variables

Age, sex, the underlying cause of end-stage renal disease, HD vintage (months), and type of vascular access were included in the data collection. Body mass index (kg/m^2^), hemoglobin (g/dL), Kt/V_urea_, serum albumin (g/dL), serum calcium (mg/dL), serum phosphorus (mg/dL), serum creatinine (mg/dL), pre-dialysis systolic blood pressure (SBP, mmHg), DBP (mmHg), and ultrafiltration volume (L/session) were collected as part of the evaluation. These data were collected monthly, and all laboratory values were averaged from the monthly collected values. Kt/V_urea_ was calculated using the Daugirdas equation [33].

Table S4 shows the medication codes. Administration of a statin for ≥30 days during 6 months of each HD quality assessment period was considered to be statin administration. The intensity of statins was classified into three groups, as previously described [34]. Patients who were administered a mean daily statin dose of <10 mg/day or an equivalent of atorvastatin were considered to have a low intensity of statin. Patients who were administered a mean daily dose ≥ 40 mg/day or an equivalent dose of atorvastatin were considered to have a high intensity of statin. Patients who received a mean daily dose between low and high intensity were considered to have a moderate intensity of statin. Consequently, we divided the patients into four groups based on the administration and intensity of statins: Group 1, patients without a prescription of statins; Group 2, patients with a prescription of a low intensity of statins; Group 3, patients with a prescription of a moderate intensity of statins; Group 4, patients with a prescription of a high intensity of statins. Medications such as aspirin, renin–angiotensin system blockades, and clopidogrel were also evaluated. If one or more prescriptions were identified for a year before the evaluation of the HD quality assessment program, it was defined as “use of the medication”.

Comorbidities were assessed for one year before the HD quality assessment program. The CCI was used to define comorbidities, and it included 17 comorbidities [35,36]. All patients in our study underwent HD and were considered to have renal disease. The CCI scores were calculated for all patients.

We followed up the patients until April 2022. The end of follow-up was the date a patient was transferred to peritoneal dialysis or kidney transplantation. The data were censored at this point. Clinical outcomes, except for death, were defined using electronic data during the follow-up period. The codes for censoring were O7072, O7071, or O7061 for peritoneal dialysis, and R3280 for kidney transplantation. Data on patient deaths were obtained from the HIRA database. 

### 4.3. Statistical Analyses

Data analysis was conducted using SAS (version 7.1) or R (version 3.5.1). Patient characteristics were summarized using frequencies and percentages for categorical data and means with standard deviations for continuous data. Differences between groups for categorical data were assessed with chi-square or Fisher’s exact tests, while ANOVA and Tukey’s test were used for continuous data. Survival curves were generated using the Kaplan–Meier method, and hazard ratios with CIs were estimated using Cox proportional hazard regression. Log-rank testing compared survival curves between groups. To account for potential influence, multivariable Cox regression analyses included demographics, comorbidities, dialysis parameters, blood values, medications, and known survival predictors. All analyses incorporated known predictors of survival as covariates. Multivariable analysis used the enter method for covariate inclusion. 

There were significant differences in baseline characteristics among the four groups. We used propensity score weighting to balance these characteristics and ensure that the results of our analyses were not biased. We created the balanced cohort for the four groups using generalized boosted models for the following variables: age; sex; body mass index; the underlying cause of end-stage renal disease; CCI score; HD vintage; ultrafiltration volume; Kt/V_urea_; hemoglobin, albumin, creatinine, phosphorus, and calcium serum levels; SBP; DBP; and the administration of aspirin, renin–angiotensin system blockade, or clopidogrel. Propensity scores were used to calculate inverse probability treatment weights. Finally, we defined the balanced cohort as a sample with weights assigned to each case. The sample size in Groups 1, 2, 3, and 4 was 52,967, 36,234, 50,524, and 32,427, respectively (Table S2). As Group 1 had the largest sample size before balancing among the four groups, the size of the weights was the smallest. Statistical significance was set at *p* < 0.05.

## 5. Conclusions

Our study exhibited significant differences in baseline characteristics between the groups, leading to limitations in establishing a robust association between statin intensity and clinical outcomes. However, we conducted various statistical analyses to mitigate these differences. Some results, including multivariable analyses controlling for baseline characteristics and analyses of a balanced cohort using propensity score weighting, indicated improved patient survival in the moderate-intensity statin group compared to non-users. These findings suggest that moderate statin use may be associated with favorable patient survival. Differences between the univariate and multivariable results in patients before balancing were mainly associated with the confounding effects of age and comorbidities. Most analyses, with adjustments for age and comorbidities, showed better survival in patients taking moderate-intensity statins than in non-users. Our results should be interpreted carefully considering the limitations of this study. Further randomized prospective studies are required to conclude whether statin intensity is associated with outcomes in patients undergoing HD.

## Figures and Tables

**Table 1:** Patient clinical characteristics.

	Group 1 (n = 37,944)	Group 2 (n = 700)	Group 3 (n = 14,160)	Group 4 (n = 541)	*p*
Age (years)	59.4 ± 13.3	62.8 ± 12.4 ^a^	62.1 ± 12.0 ^a^	62.1 ± 11.6 ^a^	<0.001
Sex (male, %)	23,450 (61.8%)	380 (54.3%)	7777 (54.9%)	304 (56.2%)	<0.001
Hemodialysis vintage (months)	56.4 ± 59.0	41.9 ± 43.1 ^a^	41.9 ± 47.4 ^a^	41.2 ± 46.6 ^a^	<0.001
Body mass index (kg/m^2^)	22.1 ± 3.3	23.4 ± 3.6 ^a^	23.3 ± 3.6 ^a^	23.5 ± 3.6 ^a^	<0.001
Underlying causes of ESRD					<0.001
Diabetes mellitus	14,642 (38.6%)	373 (53.3%)	7904 (55.8%)	340 (62.8%)	
Hypertension	10,707 (28.2%)	159 (22.7%)	3077 (21.7%)	116 (21.4%)	
Glomerulonephritis	4453 (11.7%)	58 (8.3%)	1150 (8.1%)	30 (5.5%)	
Others	3538 (9.3%)	50 (7.1%)	892 (6.3%)	25 (4.6%)	
Unknown	4604 (12.1%)	60 (8.6%)	1137 (8.0%)	30 (5.5%)	
CCI score	7.2 ± 2.9	8.1 ± 2.9 ^a^	8.2 ± 2.7 ^a^	9.1 ± 2.6 ^abc^	<0.001
Follow-up duration (months)	62.2 ± 29.2	59.2 ± 29.1 ^a^	58.5 ± 26.7 ^a^	55.9 ± 24.9 ^a^	<0.001
Type of vascular access					<0.001
Arteriovenous fistula	32,538 (85.8%)	607 (86.7%)	11,921 (84.2%)	434 (80.2%)	
Arteriovenous graft	5406 (14.2%)	93 (13.3%)	2239 (15.8%)	107 (19.8%)	
Kt/V_urea_	1.53 ± 0.27	1.53 ± 0.27	1.54 ± 0.27 ^a^	1.54 ± 0.26	0.013
Ultrafiltration volume (L/session)	2.29 ± 0.96	2.20 ± 0.94	2.23 ± 0.94 ^a^	2.35 ± 0.91 ^bc^	<0.001
Hemoglobin (g/dL)	10.6 ± 0.8	10.7 ± 0.8 ^a^	10.7 ± 0.7 ^a^	10.7 ± 0.7	<0.001
Serum albumin (g/dL)	3.99 ± 0.34	3.95 ± 0.33 ^a^	3.99 ± 0.34 ^b^	3.94 ± 0.33 ^ac^	<0.001
Serum phosphorus (mg/dL)	5.0 ± 1.4	4.8 ± 1.3 ^a^	4.9 ± 1.3 ^a^	4.7 ± 1.4 ^a^	<0.001
Serum calcium (mg/dL)	8.92 ± 0.85	8.84 ± 0.75	8.86 ± 0.76 ^a^	8.80 ± 0.76 ^a^	<0.001
Systolic blood pressure (mmHg)	141 ± 16	141 ± 16	141 ± 15	142 ± 17	0.714
Diastolic blood pressure (mmHg)	79 ± 9	77 ± 9 ^a^	77 ± 10 ^a^	74 ± 11 ^abc^	<0.001
Serum creatinine (mg/dL)	9.7 ± 2.8	9.0 ± 2.6 ^a^	9.1 ± 2.6 ^a^	8.8 ± 2.7 ^a^	<0.001
Use of RASB	11,161 (29.4%)	261 (37.3%)	4656 (32.9%)	187 (34.6%)	<0.001
Use of aspirin	13,898 (36.6%)	386 (55.1%)	8006 (56.5%)	345 (63.8%)	<0.001
Use of clopidogrel	4268 (11.2%)	174 (24.9%)	3923 (27.7%)	224 (41.4%)	<0.001
MI or CHF	15,945 (42.0%)	352 (50.3%)	7421 (52.4%)	357 (66.0%)	<0.001

Data are expressed as mean ± standard deviation for continuous variables and as numbers (percentages) for categorical variables. *p*-values are tested using one-way analysis of variance, followed by Tukey post hoc test, and Pearson’s χ^2^ test for categorical variables. Abbreviations: Group 1, patients without prescription for statins; Group 2, patients with prescription for low-intensity statins; Group 3, patients with prescription for moderate-intensity statins; Group 4, patients with prescription for high-intensity statins; CCI, Charlson comorbidity index; CHF, congestive heart failure; ESRD, end-stage renal disease; MI, myocardial infarction; RASB, renin–angiotensin system blockers. ^a^ *p* < 0.05 vs. Group 1, ^b^ *p* <0.05 vs. Group 2, ^c^ *p* <0.05 vs. Group 3.

**Table 2:** Cox regression analyses for patient survival.

	Univariate		Multivariable	
	HR (95% CI)	*p*	HR (95% CI)	*p*
Group				
Ref: Group 1				
Group 2	1.14 (1.02–1.28)	0.002	0.98 (0.85–1.12)	0.760
Group 3	1.08 (1.05–1.12)	<0.001	0.94 (0.91–0.98)	0.003
Group 4	1.25 (1.10–1.43)	<0.001	1.03 (0.87–1.22)	0.724
Ref: Group 2				
Group 3	0.95 (0.84–1.07)	0.370	0.96 (0.84–1.11)	0.597
Group 4	1.10 (0.92–1.30)	0.295	1.05 (0.85–1.30)	0.636
Ref: Group 3				
Group 4	1.16 (1.01–1.32)	0.030	1.09 (0.93–1.29)	0.294
Age (increase per 1 year)	1.06 (1.06–1.06)	<0.001	1.06 (1.06–1.06)	<0.001
Sex (ref: male)	0.87 (0.84–0.89)	<0.001	0.87 (0.84–0.89)	<0.001
Body mass index (increase in 1 kg/m^2^)	0.98 (0.97–0.98)	<0.001	0.97 (0.97–0.98)	<0.001
Underlying cause of ESRD (ref: DM)	0.81 (0.80–0.82)	<0.001	0.89 (0.88–0.91)	<0.001
Vascular access (ref: arteriovenous fistula)	1.51 (1.46–1.56)	<0.001	1.18 (1.13–1.24)	<0.001
Hemodialysis vintage (increase per 1 month)	1.00 (1.00–1.00)	0.100	1.00 (1.00–1.00)	<0.001
CCI score (increase per 1 score)	1.14 (1.13–1.14)	<0.001	1.06 (1.06–1.07)	<0.001
UFV (increase per 1 kg/session)	0.92 (0.90–0.93)	<0.001	1.08 (1.06–1.11)	<0.001
KtV_urea_ (increase per 1 unit)	0.91 (0.86–0.96)	<0.001	0.71 (0.66–0.77)	<0.001
Hemoglobin (increase per 1 g/dL)	0.87 (0.85–0.88)	<0.001	0.91 (0.89–0.93)	<0.001
Serum albumin (increase per 1 g/dL)	0.37 (0.36–0.39)	<0.001	0.63 (0.60–0.67)	<0.001
Serum creatinine (increase per 1 mg/dL)	0.87 (0.86–0.87)	<0.001	0.94 (0.93–0.95)	<0.001
Serum phosphorus (increase per 1 mg/dL)	0.85 (0.84–0.86)	<0.001	1.04 (1.03–1.06)	<0.001
Serum calcium (increase per 1 mg/dL)	0.93 (0.92–0.95)	<0.001	1.06 (1.04–1.09)	<0.001
Systolic blood pressure (increase per 1 mmHg)	1.01 (1.01–1.01)	<0.001	1.01 (1.00–1.01)	<0.001
Diastolic blood pressure (increase per 1 mmHg)	0.98 (0.98–0.99)	<0.001	1.00 (0.99–1.00)	0.066
Use of renin angiotensin system blocker	1.15 (1.12–1.18)	<0.001	1.00 (0.96–1.03)	0.952
Use of clopidogrel	1.53 (1.48–1.59)	<0.001	1.15 (1.10–1.20)	<0.001
Use of aspirin	1.16 (1.13–1.19)	<0.001	0.97 (0.93–1.00)	0.055
MI or CHF	1.49 (1.45–1.53)	<0.001	1.05 (1.01–1.09)	0.003

Multivariable analysis was adjusted for age, sex, body mass index, underlying cause of ESRD, vascular access, hemodialysis vintage, CCI score, UFV, Kt/V_urea_, hemoglobin, serum albumin, serum creatinine, serum phosphorus, serum calcium, systolic blood pressure, diastolic blood pressure, use of renin–angiotensin system blockers, statin, clopidogrel, and aspirin, MI or CHF, and was performed using enter mode. Abbreviations: Group 1, patients without prescription for statins; Group 2, patients with prescription for low-intensity statins; Group 3, patients with prescription for moderate-intensity statins; Group 4, patients with prescription for high-intensity statins; CCI, Charlson comorbidity index; CHF, congestive heart failure; CI, confidence interval; DM, diabetes mellitus; ESRD, end stage renal disease; HR, hazard ratio; MI, myocardial infarction; UFV, ultrafiltration volume.

**Table 3:** Cox regression analyses for patient survival of subgroups of MI or CVAs.

	Univariate		Multivariable	
	HR (95% CI)	*p*	HR (95% CI)	*p*
Patients with MI				
Ref: Group 1				
Group 2	1.08 (0.79–1.48)	0.619	1.23 (0.85–1.79)	0.268
Group 3	0.96 (0.88–1.04)	0.320	0.94 (0.84–1.06)	0.331
Group 4	0.92 (0.71–1.20)	0.559	1.11 (0.79–1.56)	0.541
Ref: Group 2				
Group 3	0.89 (0.65–1.21)	0.447	0.76 (0.53–1.11)	0.156
Group 4	0.85 (0.57–1.28)	0.441	0.90 (0.55–1.47)	0.675
Ref: Group 3				
Group 4	0.96 (0.74–1.26)	0.791	1.18 (0.84–1.65)	0.334
Patients with CVAs				
Ref: Group 1				
Group 2	1.12 (0.95–1.31)	0.186	1.07 (0.88–1.30)	0.505
Group 3	0.94 (0.90–0.99)	0.019	0.92 (0.86–0.98)	0.007
Group 4	0.95 (0.79–1.14)	0.600	0.97 (0.77–1.22)	0.797
Ref: Group 2				
Group 3	0.85 (0.72–0.99)	0.048	0.86 (0.70–1.05)	0.133
Group 4	0.85 (0.67–1.09)	0.199	0.91 (0.67–1.22)	0.521
Ref: Group 3				
Group 4	1.01 (0.84–1.22)	0.930	1.06 (0.84–1.33)	0.633

Multivariable analysis was adjusted for age, sex, body mass index, underlying cause of ESRD, vascular access, hemodialysis vintage, CCI score, UFV, Kt/V_urea_, hemoglobin, serum albumin, serum creatinine, serum phosphorus, serum calcium, systolic blood pressure, diastolic blood pressure, use of renin–angiotensin system blockers, statin, clopidogrel, and aspirin, MI or CHF, and was performed using enter mode. Abbreviations: Group 1, patients without prescription for statins; Group 2, patients with prescription for low-intensity statins; Group 3, patients with prescription for moderate-intensity statins; Group 4, patients with prescription for high-intensity statins; CCI, Charlson comorbidity index; CHF, congestive heart failure; CVAs, cerebrovascular accidents; CI, confidence interval; DM, diabetes mellitus; ESRD, end stage renal disease; HR, hazard ratio; MI, myocardial infarction; UFV, ultrafiltration volume.

## Data Availability

The data that support the findings of this study are available from the Health Insurance Review and Assessment Service and https://www.hira.or.kr (accessed on 9 April 2024) but restrictions apply to the availability of these data, which were used under license for the current study, and so are not publicly available. Data are however available from the authors upon reasonable request and with permission of the Health Insurance Review and Assessment Service.

## Supplementary Materials

The following supporting information can be downloaded at: https://www.mdpi.com/article/10.3390/ph17040498/s1, Figure S1: propensity score balance assessment using absolute standardized difference plot; Figure S2: Kaplan–Meier curves of patient survival according to groups using the balanced cohort; Table S1: multivariable Cox regression analyses using two variables of Group and one confounding factor; Table S2: patient clinical characteristics using the balanced cohort; Table S3: Cox regression analyses for patient survival using the balanced cohort; Table S4: Medication types and Health Insurance Review and Assessment Service codes.
