# Supplementary material for: Effects of Administration and Intensity of Statins on Mortality in Patients Undergoing Hemodialysis
Source: Pharmaceuticals (Basel). 2024 Apr 13;17(4):498. doi: 10.3390/ph17040498 (PMC11054991; doi:10.3390/ph17040498)
Supplement: Supplementary file 1 [file pharmaceuticals-17-00498-s001.zip › pharmaceuticals-2937491-supplementary.pdf]

Table S1. Multivariable Cox regression analyses using two variables of Group and one confounding factor.

Table S2. Patient clinical characteristics using the balanced cohort.

Table S3. Cox regression analyses for patient survival using the balanced cohort.

Table S4. Medication types and Health Insurance Review and Assessment Service codes.

Figure S1. Propensity score balance assessment using absolute standardized difference plot.

Figure S2. Kaplan–Meier curves of patient survival according to groups using the balanced cohort.

Table S1. Multivariable Cox regression analyses using two variables of group based on intensity of statin and one confounding factor.

	Multivariable	
	HR (95% CI)	*P*
**Analysis using group + age**		
Group 2	0.97 (0.86–1.08)	0.556
Group 3	0.95 (0.92–0.98)	<0.001
Group 4	1.10 (0.96–1.25)	0.155
**Analysis using group + VA type**		
Group 2	1.14 (1.02–1.28)	0.023
Group 3	1.07 (1.04–1.11)	<0.001
Group 4	1.22 (1.07–1.39)	0.003
**Analysis using group + CCI score**		
Group 2	0.99 (0.88–1.11)	0.881
Group 3	0.96 (0.93–0.99)	0.006
Group 4	0.99 (0.87–1.13)	0.853
**Analysis using group + use of RASB**		
Group 2	1.13 (1.01–1.27)	0.036
Group 3	1.07 (1.04–1.11)	<0.001
Group 4	1.24 (1.09–1.41)	0.001
**Analysis using group + use of clopidogrel**		
Group 2	1.08 (0.96–1.21)	0.200
Group 3	1.00 (0.97–1.04)	0.847
Group 4	1.10 (0.97–1.26)	0.153
**Analysis using group + use of aspirin**		
Group 2	1.12 (0.99–1.25)	0.063
Group 3	1.05 (1.02–1.09)	0.001
Group 4	1.21 (1.06–1.38)	0.005
**Analysis using group + presence of MI or CHF**		
Group 2	1.10 (0.98–1.24)	0.105
Group 3	1.04 (1.01–1.07)	0.014
Group 4	1.14 (1.00–1.30)	0.049
**Analysis using group + underlying disease**		
Group 2	1.06 (0.95–1.19)	0.294
Group 3	1.00 (0.97–1.03)	0.902
Group 4	1.11 (0.98–1.27)	0.113
**Analysis using group + serum albumin**		
Group 2	1.09 (0.98–1.23)	0.125
Group 3	1.07 (1.04–1.10)	<0.001
Group 4	1.16 (1.02–1.32)	0.024
**Analysis using group + serum creatinine**		

Group 2	1.04 (0.93–1.17)	0.511
Group 3	0.99 (0.96–1.02)	0.471
Group 4	1.10 (0.96–1.25)	0.165
**Analysis using group + serum phosphorus**		
Group 2	1.10 (0.98–1.23)	0.115
Group 3	1.05 (1.02–1.09)	0.001
Group 4	1.17 (1.03–1.33)	0.018
**Analysis using group + DBP**		
Group 2	1.14 (0.98–0.99)	0.033
Group 3	1.10 (1.06–1.14)	<0.001
Group 4	1.22 (1.06–1.41)	0.007

The reference group was Group 1.

Abbreviations: CCI, Charlson comorbidity index; CHF, congestive heart failure; CI, confidence interval; DBP, diastolic blood pressure; HR, hazard ratio; MI, myocardial infarction; RASB, renin-angiotensin system blocker; VA, vascular access

Table S2. Patient clinical characteristics using the balanced cohort.

	Group 1 (n = 52,967)	Group 2 (n = 36,234)	Group 3 (n = 50,524)	Group 4 (n = 32,427)	P
Age (years)	60.1 ± 0.1	61.3 ± 0.6	60.6 ± 0.1	60.7 ± 0.8	0.039
Sex (male, %)	31,791 (60.0%)	20,176 (55.7%)	29,421 (58.2%)	18,009 (55.5%)	0.230
Hemodialysis vintage (months)	52.3 ± 0.3	43.6 ± 2.2	49.3 ± 0.6	44.3 ± 3.0	<0.001
Body mass index (kg/m^2)	22.4 ± 0.0	22.6 ± 0.0	22.5 ± 0.0	22.8 ± 0.0	0.080
Underlying causes of ESRD					0.260
Diabetes mellitus	22,901 (43.2%)	18,029 (49.8%)	22,866 (45.3%)	15,924 (49.1%)	
Hypertension	14,118 (26.7%)	8,425 (23.3%)	12,781 (25.3%)	8,553 (26.4%)	
Glomerulonephritis	5,549 (10.5%)	3,689 (10.2%)	5,689 (11.3%)	2,575 (7.9%)	
Others	4,550 (8.6%)	2,792 (7.7%)	4,146 (8.2%)	1,768 (5.5%)	
Unknown	5,849 (11.0%)	3,299 (9.1%)	5,042 (10.0%)	3,607 (11.1%)	
CCI score	7.4 ± 0.0	7.7 ± 0.1	7.6 ± 0.0	7.9 ± 0.2	0.012
Follow-up duration (months)	61 ± 0	61 ± 1	60 ± 0	58 ± 2	0.638
Type of vascular access					0.446
Arteriovenous fistula	45,052 (85.1%)	31,632 (87.3%)	43,247 (85.6%)	27,458 (84.7%)	
Arteriovenous graft	7,915 (14.9%)	4,601 (12.7%)	7,275 (14.4%)	4,970 (15.3%)	
Kt/V_{urea}	1.53 ± 0.00	1.53 ± 0.01	1.54 ± 0.00	1.52 ± 0.02	0.888
Ultrafiltration volume (L/session)	2.28 ± 0.01	2.23 ± 0.04	2.24 ± 0.01	2.33 ± 0.05	0.191
Hemoglobin (g/dL)	10.7 ± 0.0	10.7 ± 0.0	10.7 ± 0.0	10.7 ± 0.0	0.121
Serum albumin (g/dL)	3.99 ± 0.00	3.99 ± 0.01	3.99 ± 0.00	3.99 ± 0.02	0.672
Serum phosphorus (mg/dL)	5.0 ± 0.0	4.9 ± 0.1	4.9 ± 0.0	5.0 ± 0.0	0.082
Serum calcium (mg/dL)	8.9 ± 0.0	8.9 ± 0.0	8.9 ± 0.0	8.9 ± 0.0	0.611
Systolic blood pressure (mmHg)	141 ± 0	141 ± 1	141 ± 0	142 ± 1	0.747
Diastolic blood pressure (mmHg)	78 ± 0	78 ± 0	78 ± 0	78 ± 1	0.470
Serum creatinine (mg/dL)	9.5 ± 0.0	9.4 ± 0.1	9.4 ± 0.0	9.4 ± 0.2	0.227
Use of RASB	16,031 (30.3%)	12,755 (35.2%)	16,320 (32.3%)	12,489 (38.5%)	0.020

Use of clopidogrel	8,311 (15.7%)	6,731 (18.6%)	8,649 (17.1%)	6,830 (21.1%)	0.028
Use of aspirin	22,264 (42.0%)	17,127 (47.3%)	22,350 (44.2%)	15,786 (48.7%)	0.070
MI or CHF	23,686 (44.7%)	17,470 (48.2%)	23,321 (46.2%)	15,827 (48.8%)	0.329

Data are expressed as mean ± standard errors for continuous variables and as numbers (percentages) for categorical variables. *P*-values are tested using one-way analysis of variance and Pearson's χ^2 test for categorical variables.

Abbreviations: Group 1, patients without prescription for statins; Group 2, patients with prescription for low-intensity statins; Group 3, patients with prescription for moderate-intensity statins; Group 4, patients with prescription for high-intensity statins; CCI, Charlson comorbidity index; CHF, congestive heart failure; ESRD, end-stage renal disease; MI, myocardial infarction; RASB, renin-angiotensin system blockers.

Table S3. Cox regression analyses for patient survival using the balanced cohort.

	Univariate		Multivariable	
	HR (95% CI)	*P*	HR (95% CI)	*P*
Group				
Ref: Group 1				
Group 2	0.95 (0.93–0.97)	<0.001	0.95 (0.92–0.97)	<0.001
Group 3	0.96 (0.94–0.98)	<0.001	0.93 (0.91–0.95)	<0.001
Group 4	1.05 (1.03–1.08)	<0.001	1.00 (0.97–1.03)	0.939
Ref: Group 2				
Group 3	1.02 (0.99–1.04)	0.182	0.98 (0.95–1.00)	0.107
Group 4	1.11 (1.08–1.14)	<0.001	1.06 (1.02–1.09)	<0.001
Ref: Group 3				
Group 4	1.09 (1.07–1.12)	<0.001	1.08 (1.05–1.11)	<0.001
Age (increase per 1 year)	1.06 (1.06–1.06)	<0.001	1.06 (1.06–1.06)	<0.001
Sex (ref: male)	0.82 (0.81–0.83)	<0.001	0.78 (0.77–0.80)	<0.001
Body mass index (increase 1 kg/m^2)	0.97 (0.97–0.97)	<0.001	0.96 (0.96–0.97)	0.005
Underlying cause of ESRD (ref: DM)	0.82 (0.82–0.83)	<0.001	0.87 (0.86–0.88)	<0.001
Vascular access (ref: arteriovenous fistula)	1.34 (1.31–1.36)	<0.001	1.16 (1.13–1.18)	<0.001
Hemodialysis vintage (increase per 1 month)	1.01 (1.01–1.01)	<0.001	1.00 (1.00–1.01)	<0.001
CCI score (increase per 1 score)	1.14 (1.14–1.14)	<0.001	1.08 (1.07–1.08)	<0.001
Ultrafiltration volume (increase per 1 kg/session)	0.92 (0.91–0.92)	<0.001	1.06 (1.05–1.07)	<0.001
KtV_{urea} (increase per 1 unit)	0.91 (0.88–0.94)	<0.001	0.66 (0.63–0.69)	<0.001
Hemoglobin (increase per 1 g/dL)	0.87 (0.86–0.88)	<0.001	0.93 (0.92–0.94)	<0.001
Serum albumin (increase per 1 g/dL)	0.39 (0.38–0.40)	<0.001	0.66 (0.64–0.68)	<0.001
Serum creatinine (increase per 1 mg/dL)	0.88 (0.87–0.88)	<0.001	0.95 (0.95–0.96)	<0.001
Serum phosphorus (increase per 1 mg/dL)	0.90 (0.89–0.91)	<0.001	1.07 (1.06–1.07)	<0.001
Serum calcium (increase per 1 mg/dL)	1.01 (0.99–1.02)	0.063	1.13 (1.12–1.14)	<0.001
Systolic blood pressure (increase per 1 mmHg)	1.01 (1.01–1.01)	<0.001	1.01 (1.01–1.01)	<0.001

Diastolic blood pressure (increase per 1 mmHg)	0.98 (0.98–0.98)	<0.001	0.99 (0.99–0.99)	0.009
Use of renin angiotensin system blocker	1.20 (1.18–1.21)	<0.001	1.05 (1.03–1.07)	<0.001
Use of clopidogrel	1.54 (1.51–1.56)	<0.001	1.18 (1.15–1.21)	<0.001
Use of aspirin	1.24 (1.22–1.25)	<0.001	1.00 (0.99–1.02)	0.640
MI or CHF	1.40 (1.38–1.43)	<0.001	1.03 (1.01–1.05)	<0.001

Multivariable analysis was adjusted for age, sex, body mass index, underlying cause of ESRD, vascular access, hemodialysis vintage, CCI score, ultrafiltration volume, Kt/V_{urea}, hemoglobin, serum albumin, serum creatinine, serum phosphorus, serum calcium, systolic blood pressure, diastolic blood pressure, use of renin–angiotensin system blockers, statin, clopidogrel, and aspirin, MI or CHF, and was performed using enter mode.

Abbreviations: Group 1, patients without prescription for statins; Group 2, patients with prescription for low-intensity statins; Group 3, patients with prescription for moderate-intensity statins; Group 4, patients with prescription for high-intensity statins; CCI, Charlson comorbidity index; CHF, congestive heart failure; CI, confidence interval; DM, diabetes mellitus; ESRD, end stage renal disease; HR, hazard ratio; MI, myocardial infarction.

Table S4. Medication types and Health Insurance Review and Assessment Service codes

Medications	Codes
**Acepril**	104201ATB, 104202ATB
**Amlodipine**	495901ATB, 459802ACH, 483201ATB, 486501ATB, 107601ATB, 107601ATD, 459801ACH, 459801ATB, 459901ATB, 464601ATB, 470801ATB, 476201ATB, 479701ATB, 483202ATB, 486502ATB, 107602ATB, 107602ATD, 470802ATB
**Amlodipine+Atorvastatin**	614500ATB, 472300ATB472400ATB472500ATB518900ATB
**Amlodipine+Losartan+Chlorthalidone**	662800ATB, 662900ATB, 663000ATB
**Amlodipine+Losartan+Rosuvastatin**	663900ATB, 664000ATB, 664100ATB, 664200ATB, 664300ATB, 664400ATB
**Amlodipine+Olmesartan+Rosuvastatin**	677300ATB, 677400ATB, 677500ATB, 677600ATB
**Amlodipine+Rosuvastatin**	673900ATB, 674000ATB, 674100ATB
**Amlodipine+Rosuvastatin+Telmisartan**	671200ATB, 671300ATB, 671400ATB, 671500ATB, 677000ATB, 677100ATB, 671600ATB, 671700ATB
**Amlodipine+Telmisartan+Hydrochlorothiazide**	663500ATB, 663600ATB, 663700ATB, 663800ATB
**Amosulalol**	107901ATB, 107902ATB
**Arotinolol**	110202ATB, 110201ATB
**Atenolol**	483102ATB, 111402ATB, 483101ATB, 111403ATB, 111401ATB
**Atenolol+Chlorthalidone**	262100ATB, 460200ATB
**Azilsartan**	662401ATB, 662403ATB, 662402ATB
**Azilsartan+Chlorthalidone**	673500ATB, 673600ATB
**Barnidipine**	114003ACH, 114001ACH, 114002ACH
**Benidipine**	115101ATB, 115102ATB, 115104ATB, 115103ATB
**Betaxolol**	116801ATB, 116803ATB
**Bevantolol**	117002ATB, 117001ATB
**Bisoprolol**	117904ATB, 117903ATB, 117902ATB, 117901ATB
**Bisoprolol+Hydrochlorothiazide**	469800ATB, 470000ATB, 469900ATB

**Candesartan**	122601ATB, 122602ATB, 122603ATB
**Candesartan+Amlodipine**	652900ATB, 653000ATB, 653100ATB
**Candesartan+Hydrochlorothiazide**	423700ATB
**Candesartan+Rosuvastatin**	661800ATB, 661900ATB, 673700ATB, 662000ATB, 662100ATB
**Captopril**	122901ATB, 122902ATB, 122903ATB
**Captopril+Hydrochlorothiazide**	262200ATB, 262300ATB
**Carteolol**	124801ATB
**Carvedilol**	125005ATB, 125003ATB, 662201ATB, 125008ACR, 125001ATB, 662202ATB, 125007ACR, 125002ATB, 125006ACR, 125004ACR
**Celiprolol**	129101ATB
**Cilazapril**	133001ATB, 133002ATB, 133003ATB
**Cilnidipine**	133102ATB, 133101ATB
**Clonidine**	136505ATR
**Diltiazem**	145706ATB, 145707ACR, 145707ATR, 145703ACR, 145706ATR, 145707ATB
**Doxazocin**	149101ATB, 149102ATB, 149104ATR, 149103ATB
**Efonidipine**	441202ATB, 441201ATB
**Enalapril**	151603ATB, 151601ATB
**Enalapril+Hydrochlorothiazide**	453700ATB, 440300ATB
**Eprosartan**	429201ATB
**Eprosartan+Hydrochlorothiazide**	460500ATB
**Felodipine**	157503ATR, 157501ATR
**Felodipine+Metoprolol**	262400ATR
**Fimasartan**	515203ATB, 515201ATB, 515202ATB
**Fimasartan+Amlodipine**	651900ATB, 652000ATB, 652700ATB, 652100ATB
**Fimasartan+Hydrochlorothiazide**	522000ATB, 526800ATB
**Fimasartan+Rosuvastatin**	655000ATB, 654900ATB, 654800ATB, 654700ATB, 654600ATB
**Fosinopril**	163501ATB, 163502ATB

**Hydralazine**	170701ATB
**Imidapril**	173402ATB, 173401ATB
**Irbesartan**	177301ATB, 177303ATB
**Irbesartan+Atorvastatin**	524000ATB, 524100ATB, 527100ATB, 527000ATB
**Irbesartan+Hydrochlorothiazide**	385700ATB, 385800ATB, 553800ATB
**Lacidipine**	180301ATB, 180302ATB, 180303ATB
**Lercanidipine**	182001ATB, 182002ATB
**Lisinopril**	184501ATB
**Lisinopril+Hydrochlorothiazide**	499200ATB, 499300ATB
**Losartan**	185701ATB, 185702ATB
**Losartan+Amlodipine**	503000ATB, 637400ATB, 513900ATB, 637500ATB, 502700ATB, 637600ATB
**Losartan+Hydrochlorothiazide**	262500ATB, 486900ATB, 378900ATB
**Manidipine**	188001ATB, 188002ATB
**Metoprolol**	194003ATR, 193802ATB, 262400ATR
**Metoprolol+Hydrochlorothiazide**	262600ATB
**Metoprolol+felodipine**	262400ATR
**Minoxidil**	196102ATB
**Nadolol**	198301ATB
**Nebivolol**	489501ATB, 489502ATB, 489503ATB
**Nicardipine**	201003ACR, 201002ATB
**Nifedipine**	201407ACS, 201405ATR, 528201ATR, 201409ATR, 528202ATR, 201401ACS, 201401ATB, 201408ATR
**Nimodipine**	201901ATB, 356202ATR, 356203ATR, 356201ATB, 356202ATB
**Nisoldipine**	356202ATR
**Olmesartan**	468502ATB, 468501ATB, 468503ATB, 520902ATB, 520901ATB

**Olmesartan+Amlodipine**	547800ATB, 632800ATB, 500500ATB, 547700ATB, 629500ATB, 631300ATB, 500600ATB, 547900ATB, 632900ATB, 547600ATB, 548000ATB, 582200ATB, 629600ATB, 633000ATB, 547500ATB, 582400ATB, 629400ATB
**Olmesartan+Amlodipine+Hydrochlorothiazide**	519800ATB, 519700ATB, 520100ATB, 520000ATB, 519900ATB
**Olmesartan+Hydrochlorothiazide**	513600ATB, 489100ATB
**Olmesartan+Rosuvastatin**	644200ATB, 644100ATB, 526900ATB, 526300ATB, 526400ATB, 653200ATB, 526500ATB
**Perindopril**	211301ATB, 501601ATB, 211302ATB, 501602ATB
**Perindopril+Indapamide**	556200ATB
**Propranolol**	219901ATB, 219904ATB, 219906ACR, 219905ACR
**Quinapril**	221901ATB
**Ramipril**	222401ATB, 222402ATB, 222404ATB
**Ramipril+Felodipine**	447100ATB, 447200ATB
**Ramipril+Hydrochlorothiazide**	448600ATB, 448700ATB
**Telmisartan**	378801ATB, 378802ATB, 378803ATB
**Telmisartan+Amlodipine**	521200ATB, 511600ATB, 521300ATB, 511700ATB, 521400ATB, 511500ATB, 644800ATB, 623100ATB
**Telmisartan+Hydrochlorothiazide**	443200ATB, 443300ATB, 502600ATB
**Telmisartan+Rosuvastatin**	631600ATB, 629900ATB, 630000ATB, 631700ATB, 630100ATB, 630200ATB
**Temocapril**	235002ATB
**Terazosin**	235501ATB, 235502ATB, 235503ATB, 616501ATB
**Valsartan**	247103ATB, 247101ATB, 247102ATB, 247104ATB
**Valsartan+Amlodipine**	522600ATB, 492900ATB, 522900ATB, 523200ATB, 522700ATB, 492800ATB, 522800ATB, 523000ATB, 523300ATB, 495800ATB, 523100ATB, 523400ATB
**Valsartan+Hydrochlorothiazide**	356400ATB, 442600ATB
**Valsartan+Lercanidipine**	522200ATB, 522300ATB, 522400ATB
**Valsartan+Pitavastatin**	635000ATB, 635200ATB, 634900ATB, 635100ATB

**Valsartan+Rosuvastatin**	629700ATB, 525000ATB, 525200ATB, 629800ATB, 525100ATB, 525300ATB
**Valsartan+Sacubitril**	651401ATB, 651402ATB, 651403ATB
**Verapamil**	247606ATB, 247607ATB, 247603ATR, 247605ATR, 247601ACR
**Zofenopril**	510401ATB, 510402ATB, 510403ATB
**Atorvastatin+Amlodipine**	472300ATB, 472400ATB
**Atorvastatin+Ezetimibe**	633800ATB, 633900ATB, 634800ATB
**Pitavastatin+Fenofibrate**	679300ACH
**Rosuvastatin+Ezetimibe**	640700ATB, 640800ATB, 640900ATB
**Rosuvastatin+Ezetimibe+Telmisartan**	671400ATB, 671500ATB, 671700ATB
**Aspirin**	110701ATB, 110702ATB, 110801ATB, 110802ATB, 111001ACE, 111001ATB, 111001ATE, 111002ATE, 111003ACE, 111003ATE
**Clopidogrel**	133201ACR, 133201ATB, 133201ATR, 133202ATB, 133203ATR, 506100ATB
**Cilostazol**	136901ATB, 492501ATB, 495201ATB, 498801ATB, 501501ATB
**Ticlopidine**	498900ATB, 239201ATB, 239202ATB
**Aspirin+Bethocarbamol**	256800ATB
**Aspirin+Clopidogrel**	517900ACH, 517900ACE, 517900ATE, 667500ACE
**Aspirin+Dipyridamole**	489700ACR
**Atorvastatin 10mg**	111501ATB, 502201ATB, 633800ATB, 472300ATB, 524000ATB, 527100ATB, 614500ATB, 671800ATR, 671900ATR, 673800ATR
**Atorvastatin 20mg**	111502ATB, 502202ATB, 633900ATB, 472400ATB, 518900ATB, 524100ATB, 527000ATB, 672000ATR, 672100ATR
**Atorvastatin 40mg**	111503ATB, 502203ATB, 634800ATB, 472500ATB,
**Atorvastatin 80mg**	111504ATB, 502204ATB
**Fluvastatin 20mg**	162401ACH
**Fluvastatin 40mg**	162402ACH
**Fluvastatin 80mg**	162403ATR
**Lovastatin 20mg**	185801ATB

**Pitavastatin 1mg**	470902ATB
**Pitavastatin 2mg**	470901ATB, 634900ATB, 635000ATB
**Pitavastatin 4mg**	470903ATB, 635100ATB, 635200ATB
**Pravastatin 5mg**	216602ATB
**Pravastatin 10mg**	216601ATB
**Pravastatin 20mg**	216603ATB
**Pravastatin 40mg**	216604ATB, 519300ACH
**Rosuvastatin 5mg**	454003ATB, 640700ATB, 663400ACS, 526900ATB, 629700ATB, 629800ATB, 631600ATB, 631700ATB, 644200ATB, 654800ATB, 655000ATB, 661800ATB, 663900ATB, 664200ATB, 671200ATB, 671300ATB, 664600ATB, 631600ATB, 631700ATB, 673700ATB, 673900ATB, 672500ATR, 672600ATR,
**Rosuvastatin 10mg**	454001ATB, 640800ATB, 525000ATB, 525100ATB, 526300ATB, 629900ATB, 630100ATB, 644100ATB, 653200ATB, 654700ATB, 654900ATB, 661900ATB, 662000ATB, 664000ATB, 664300ATB, 671400ATB, 671500ATB, 671600ATB, 664700ATB, 674000ATB, 672700ATR, 672800ATR
**Rosuvastatin 20mg**	454002ATB, 640900ATB, 525200ATB, 525300ATB, 526400ATB, 526500ATB, 630000ATB, 630200ATB, 654600ATB, 622100ATB, 664100ATB, 664400ATB, 671700ATB, 664800ATB, 674100ATB, 672900ATR, 673000ATR
**Simvastatin 5mg**	227806ATB
**Simvastatin 10mg**	471000ATB, 227803ATB,
**Simvastatin 20mg**	227801ATB, 227801ATR, 471100ATB, 631400ATB,
**Simvastatin 40mg**	227802ATB, 507800ATB, 631500ATB
**Simvastatin 80mg**	227805ATB

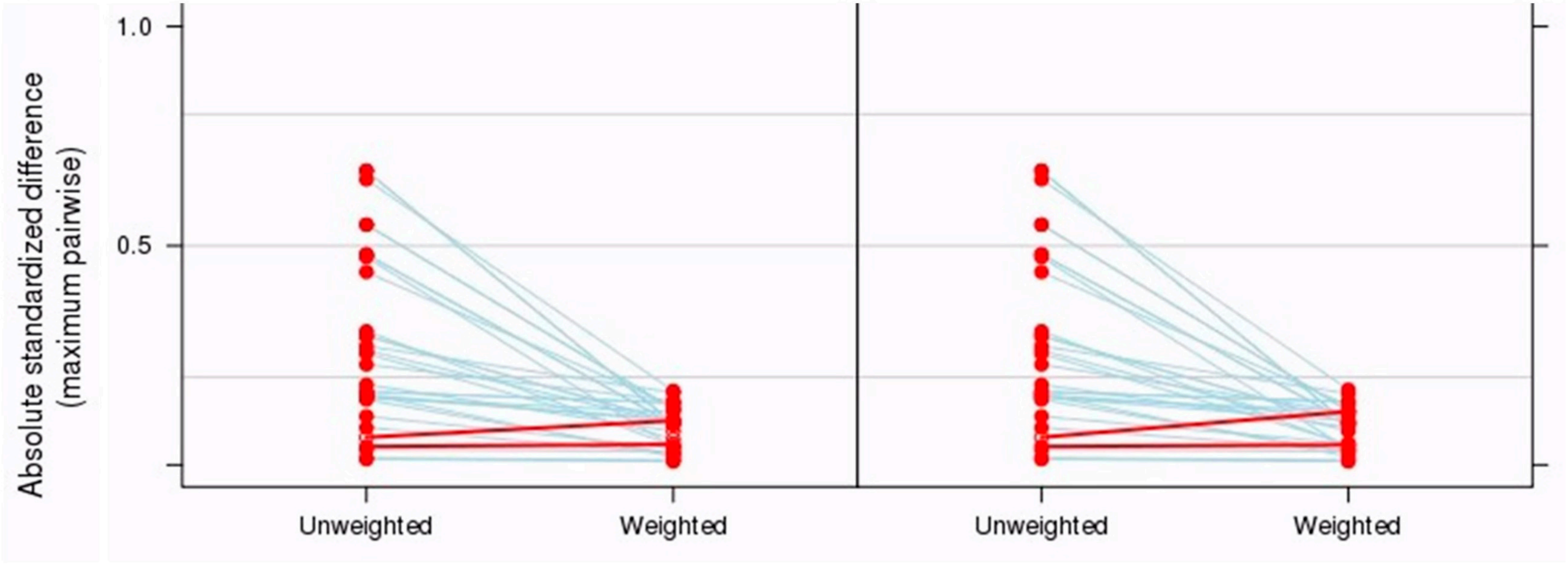

Figure S1. Propensity score balance assessment using absolute standardized difference plot. Left: effect size. Right: Kolmogorov–Smirnov statistics.

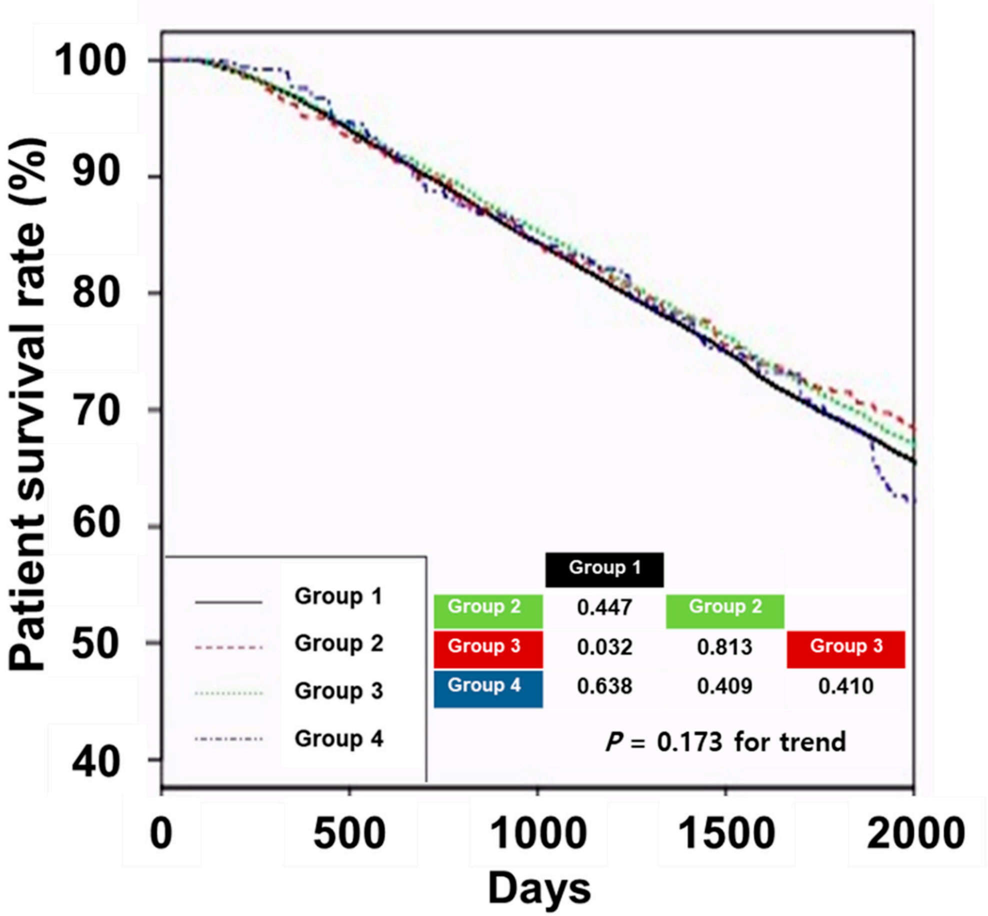

Figure S2. Kaplan–Meier curves of patient survival according to groups using the balanced cohort. The 5-year survival rates in Groups 1, 2, 3, and 4 were 68.7%, 71.5%, 70.2%, and 68.5%, respectively. The *P*-values for pairwise comparison or trend with log-rank tests were added to the lower right corner of the graph.
